# Vector Competence: What Has Zika Virus Taught Us?

**DOI:** 10.3390/v11090867

**Published:** 2019-09-17

**Authors:** Sasha R. Azar, Scott C. Weaver

**Affiliations:** 1 Department of Microbiology and Immunology, University of Texas Medical Branch, 300 University Blvd, Galveston, TX 77555, USA; 2 Institute for Translational Sciences, University of Texas Medical Branch, 300 University Blvd, Galveston, TX 77555, USA; 3 Institute for Human Infections and Immunity, University of Texas Medical Branch, 300 University Blvd, Galveston, TX 77555, USA

**Keywords:** Zika virus, mosquitoes, vector competence, arbovirus, *Aedes aegypti*, Flaviviruses

## Abstract

The unprecedented outbreak of Zika virus (ZIKV) infection in the Americas from 2015 to 2017 prompted the publication of a large body of vector competence data in a relatively short period of time. Although differences in vector competence as a result of disparities in mosquito populations and viral strains are to be expected, the limited competence of many populations of the urban mosquito vector, *Aedes aegypti*, from the Americas (when its susceptibility is viewed relative to other circulating/reemerging mosquito-borne viruses such as dengue (DENV), yellow fever (YFV), and chikungunya viruses (CHIKV)) has proven a paradox for the field. This has been further complicated by the lack of standardization in the methodologies utilized in laboratory vector competence experiments, precluding meta-analyses of this large data set. As the calls for the standardization of such studies continue to grow in number, it is critical to examine the elements of vector competence experimental design. Herein, we review the various techniques and considerations intrinsic to vector competence studies, with respect to contemporary findings for ZIKV, as well as historical findings for other arboviruses, and discuss potential avenues of standardization going forward.

## 1. Introduction

Zika virus (ZIKV) is an arthropod-borne virus (arbovirus) of the genus *Flavivirus* and family *Flaviviridae*. It was originally discovered in 1947 from the blood of a febrile sentinel rhesus macaque placed in a treetop platform in the Ziika forest in Uganda [1,2]. Early characterization, as well as the infection of a single human volunteer, suggested that infection produces minimal disease [2,3]. Seroprevalence analyses also indicate that ZIKV has circulated in Southeast Asia as early as the 1950s [4,5,6], although it was not associated with reports of human illness. Similar to the related flaviviruses yellow fever (YFV) and dengue (DENV), ZIKV demonstrates evidence of an African enzootic, or sylvatic transmission cycle, circulating between forest canopy-dwelling mosquitoes such as *Aedes* (*Stegomyia*) *africanus* Theobald or *Aedes* (*Diceromyia*) *furcifer* Edwards and nonhuman primates such as patas or African green monkeys [7,8,9,10,11].

Beginning in 2007, Zika began to emerge outside of endemic areas in a series of outbreaks beginning on the Island of Yap in the Federated States of Micronesia, and in Gabon [12,13]. Following these outbreaks, ZIKV spread across the South Pacific, causing epidemics in French Polynesia, New Caledonia, Easter Island, and the Cook Islands [14,15], before reaching the Americas (Brazil) proper in 2013 [16,17,18]. There, the virus was initially undetected, which was possibly due to it being overshadowed by an outbreak of chikungunya virus (CHIKV) [19]. During the outbreaks taking place in the South Pacific, the capacity for ZIKV to cause some of the unique clinical outcomes and sequalae (e.g., Guillain–Barré syndrome, congenital malformations, and shedding of the virus in several biological fluids/sexual transmission) was first appreciated, or subsequently elucidated via retrospective analyses [20,21]. The severity of these sequalae, as well as the explosivity of the spread in the Americas (reaching estimates of between 400,000–1,300,000 infected individuals in Brazil alone, and emergence/spread into 50 countries or territories in the Americas by March of 2016) [22] led the World Health Organization to declare the ongoing transmission of ZIKV in the Americas to be a public health emergency of international concern, which was a status that had been rescinded as the outbreak began to taper in the later summer and fall of 2016 [21]. Substantial efforts were rapidly undertaken for the development of ZIKV vaccines and therapeutics [23], culminating in several vaccine candidates reaching various stages of clinical testing [24,25,26]. Despite the relatively expeditious advancement of these candidates, none has yet overcome the hurdle of clinical licensure; as such, the best methodologies for combating ZIKV outbreaks remain public education and mosquito abatement.

The breadth of the 2015–2017 ZIKV outbreak was unprecedented in the amount of information that became available very quickly. Currently, there are over 5000 publications on “Zika virus” indexed in PubMed, compared to 55 indexed as of 2010. Of these publications, a large number has explored the vector competence of various populations of potential vector mosquitoes to multiple strains of ZIKV using various experimental approaches. Collectively, these efforts are best summarized by the finding that susceptibility to infection, and ability to transmit ZIKV varies widely across disparate geographic strains of several mosquito species, and disparate strains of ZIKV also vary greatly in their ability to be transmitted [27,28,29]. Similar findings have been widely accepted with respect to vector competence of multiple arboviruses [27]. Despite this typical variability, the principal ZIKV vector, *Aedes aegypti,* has often proved relatively refractory to infection, especially in the context of viral titers representative of human viremias [27,29,30]. However, drawing overarching conclusions from this vast accumulation of data is difficult, largely due to disparities in the methodologies that have been used to assess vector competence, precluding meta-analyses. To address this problem, there have been various calls for the standardization of techniques, reporting, and methodologies in laboratory arthropod infection studies. As a first step to this end, we examine here the methodologies, techniques, and considerations intrinsic to vector competence analyses, utilizing the body of ZIKV literature as an example, and making comparisons with previously published work on other mosquito-borne arboviruses.

## 2. Vectorial Capacity and Vector Competence: Definitions and Considerations

**Figure 1:**
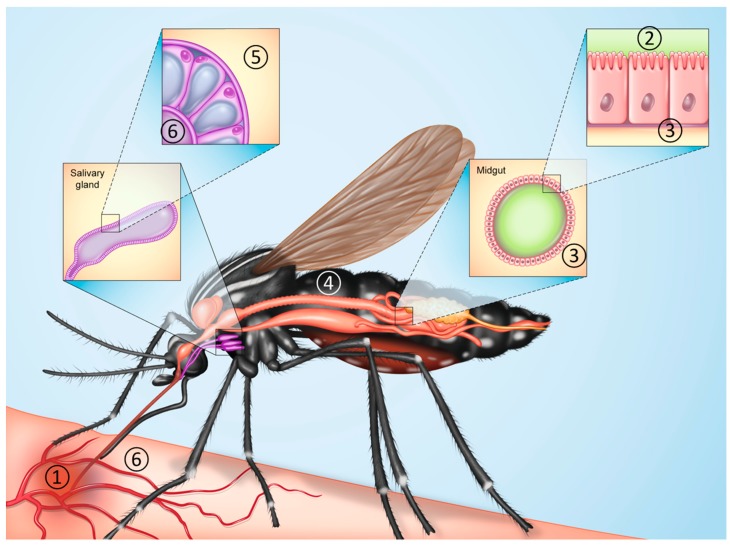
Sequential steps required for a female mosquito to transmit a virus biologically. (**1**) The virus in question is imbibed by a female mosquito from an viremic host, (**2**) the virus infects and replicates in midgut epithelial cells (overcoming the midgut infection barrier), (**3**) the virus escapes from midgut epithelial cells into the mosquito’s hemolymph within the hemocoel (overcoming the midgut escape barrier to develop a disseminated infection), (**4**) the infection of and replication in peripheral tissues/organs such as nerves, muscle fibers, or fat body, (**5**) the infection of salivary glands (this step can potentially occur in the absence of infection/replication of peripheral tissues/organs, overcoming the salivary gland infection barrier), and (**6**) shedding into the apical cavities of acinar cells and presence in the saliva for inoculation into subsequent hosts upon feeding [31,34,35]. Vector Competence Outlined.

In any discussion of vector competence, delineating the differences vis-a-vis vectorial capacity is critical. Vector competence is disproportionately overrepresented in the modern literature, and is simply defined as the ability for a given vector to acquire and subsequently transmit a pathogen [27] (summarized in Figure 1). For mosquito-borne arboviruses, the virus is initially imbibed by an adult female from a viremic host (Figure 1, step 1). The first major hurdle the virus faces is the midgut infection barrier (MIB), in which the virus must infect the epithelial cells of the midgut, overcoming digestive proteolytic enzymes, RNA interference (RNAi), effects of the luminal and sometimes internal microbiota, the formation of the peritrophic matrix, as well the physical barrier represented by the midgut epithelium itself [31] (Figure 1, step 2). From this point, the virus must undergo replication in the midgut epithelial cells, be shed from its basolateral aspect, and traverse the basal lamina into the hemolymph (Figure 1, step 3). Failure of the virus to escape or disseminate from an infected midgut is called the midgut escape barrier (MEB). During this stage of the infectious process, the virus may infect and replicate in secondary tissues such as the fat body, hemocytes, nerves, and muscles (Figure 1, step 4), although such infection of secondary tissues may not be strictly required. The salivary gland infection barrier (SGIB) represents the next step in the progression of vector competence, and can be considered an extension of dissemination from the midgut (disseminated infection). The basal lamina surrounding this organ must first be traversed (Figure 1, step 5). Upon infection of the salivary gland acinar cells of a competent vector, the virus is subsequently shed from the acinar cells into apical cavities, such that it is inoculated with saliva into subsequent hosts upon blood feeding (Figure 1, step 6). The time period between oral exposure to the virus and the presence of virus in the saliva is known as the extrinsic incubation period, or EIP. Although rare, reports do exist in which a virus that is capable of infecting salivary glands is incapable of being shed efficiently into saliva [32], indicating the existence of a salivary gland escape barrier [27,31,33,34,35] (SGEB). Generally, the presence of virus in the saliva extends for the life of the mosquito. In the laboratory settings, vector competence is most synonymous with transmission efficiency (viral deposition in saliva), although competence and refractoriness are often estimated based on midgut infection and dissemination into the hemocoel, assuming the lack of salivary gland barriers, which are relatively rare.

Although both terms are often used interchangeably, the vectorial capacity is a far more complex parameter, and is defined as:

(1) $$Vectorial\;Capacity\;\left(VC\right)=ma^{2}bp^{n}/-log_{e}p$$

where *m* = the number of female mosquitoes with respect to the host, *a* = the daily blood-feeding rate on the host in question, *b* = the transmission rate among exposed mosquitoes (vector competence), *p* = the daily survival rate of the mosquito species/population in question, and *n* = the time it takes for the mosquito species/population in question to transmit the virus after initial exposure (EIP) [27,31,36]. Vectorial capacity encompasses a marriage between mosquito intrinsic factors (genotype, microbiome, etc.) as well as extrinsic factors, often environmental, that affect behavior, longevity, etc. Indeed, vector competence itself encompasses two of the parameters that inform a vector’s capacity—“b” the transmission rate, and “n” the EIP; both can be empirically determined for a given mosquito population and agent in laboratory settings. In considering what role vectorial capacity plays in anticipating/addressing arboviral outbreaks, one can consider the mosquito species *Aedes albopictus* (Skuse). Prior to the Indian Ocean Lineage (IOL) chikungunya virus outbreaks taking place beginning in 2005, *Ae. albopictus* was largely considered to be a secondary vector for a multitude of arboviral pathogens, which was a tenant that was at least in part driven by its more catholic feeding behavior compared to the highly anthropophilic *Ae. aegypti* [37,38,39,40,41]. These more indiscriminate feeding behaviors, coupled with its ecologic plasticity and the field isolations of a multitude of arboviral agents, both zoonotic and mainly human, from pools of *Ae. albopictus* (including but not limited to La Crosse, eastern equine encephalitis, and Japanese encephalitis viruses [39,40]) have largely supported its preconceived role as a secondary or “bridge” vector. Nonetheless, in the absence of *Ae. aegypti* (e.g., reports of autochthonous dengue transmission in Suffolk County New York in 2013 [42], as well as the outbreaks of CHIKV in Italy and France in 2017 [43,44,45]), or in the face of overwhelming field evidence (e.g., ZIKV-positive, DENV2-positive, and CHIKV-positive *Ae. albopictus* pools from the 2007 outbreaks in Gabon [13,46]), this species can serve as a primary epidemic vector. Furthermore, even poorly competent/relatively refractory vectors are capable of initiating/sustaining outbreaks of arboviral disease if behavioral and ecologic factors, which play major roles in vectorial capacity, compensate [47,48,49].

## 3. Virological Elements

Laboratory vector competence determinations require a marriage between the underlying microbiology and entomology. Therefore, to properly design and execute vector competence analyses for arboviruses, some consideration of the virological underpinnings are critical. Considered here are the contributions of viral evolution/genotype/strain, storage/freshness, identity as an isolate or as part of an infectious clone-derived population, and cell culture passage history.

### 3.1. Effect of Arboviral Evolution on Vector Competence

Upon the introduction of ZIKV into the Americas, two hypotheses gained considerable traction as potential explanations for the unprecedented breadth of the outbreak and the observation of sequelae such as congenital Zika syndrome (CZS) and Guillain–Barré syndrome: i) ZIKV underwent an adaptive evolutionary process that enhanced urban transmission by either adapting for enhanced mosquito infectivity/transmission, or increasing/prolonging human viremias, and/or ii) the virus was stochastically introduced to a region with a large immunologically naïve population with a large population of *Ae. aegypti* mosquitoes, allowing for both explosive transmission and the emergence of rare sequelae [50]. Of these hypotheses, laboratory vector competence evaluations allow for the direct examination of whether ZIKV had adapted for more efficient transmission by urban vectors such as *Ae. aegypti*. Given that ZIKV exists as two distinct lineages, African and Asian, with the American outbreak strain being considered a recently evolved clade within the Asian lineage [51], vector competence analyses were able to examine not only whether recent strains are more transmissible, but also whether this was associated with lineage/genotype. Surprisingly, several early studies demonstrated that multiple *Ae. aeygpti* populations were more efficiently infected by African lineage ZIKV compared to Asian lineage or American outbreak strains [29,52,53,54], which was a finding that was also demonstrated in one population of *Ae. albopictus* [28]. These data appear to indicate that the contemporary strains from the Asian lineage and the American outbreaks are not more infectious in urban mosquito populations, although mosquito population combinations lead to a high degree of variation in experimental outcomes. This finding is best exemplified by one analysis that noted strain variation in terms of infectivity and transmissibility within the African ZIKV clade in a single population of mosquitoes (*Ae. aegypti*, New Caledonia) [52]. Nonetheless, the unprecedented spread of the American outbreak and the lack of major outbreaks detected in Asia [4] led some to investigate differences in transmissibility between American outbreak strains and other Asian lineage strains. So far, only one hypothesis that has been supported as related to NS1 antigenemia, which was associated with an A188V mutation in Asian lineage ZIKV strains just before the South Pacific and American outbreaks began. The increased NS1 secretion from infected host cells mediated by this substitution, and thereby a higher antigen load, enhances Ae. aegypti infectivity, which may have contributed to ZIKV’s recent efficient spread [55].

The genotypic variability of arboviruses in experimental vector infection/transmission is an oft-reported finding in the vector competence literature. For example, the NY99 genotype of West Nile virus (WNV) that was introduced in the United States was later displaced by a new genotype called WN02, which was characterized by a single amino acid substitution in the envelope protein (V159A) [56,57,58]. This single envelope mutation was found to mediate a substantially shortened EIP in laboratory vector competence analyses in both *Culex pipiens* and *Cx. tarsalis* [56,57], abruptly shifting the vectorial capacity for this genotype compared to NY99 strains. In addition, experimental evidence suggests that the major enzootic host, the house sparrow (*Passer domesticus*), developed resistance to NY99 lineage WNV viremia over time, which taken in the context of the higher peak viremias observed in the WN02 and SW03 genotypes, was posited as contributing to the NY99 genotype’s disappearance [59]. Likewise, Venezuelan equine encephalitis virus (VEEV) exists as seven different species and multiple subtypes. Within subtype I (the most characterized subtype), there exist both enzootic (Subtypes ID, IE, IF), and epizootic (IAB, IC) serotypes. Enzootic VEEV viruses circulate predominantly between *Culex* (*Melanoconion*) spp. vectors and rodent reservoir hosts, and go largely unnoticed as human pathogens, because most infections are easily confused with dengue in the absence of laboratory diagnostics [60,61]. Mutations in the E2 protein of sylvatic precursors allow for the significant amplification of VEEV in equids, and are additionally associated with shifts in vectors toward *Aedes taeniorhynchus* and *Psorophora* spp., which tend to bite large mammals such as horses, leading to epizootic outbreaks/transmission [60,62,63,64,65]. Ultimately, such considerations in viral evolution and lineage in vector competence experiments are highly contingent on the experimental questions or hypotheses at play.

### 3.2. History of Virus Stocks used for Bloodmeal Preparation

In addition to the role of the virus strain/genotype, some consideration toward experimental bloodmeal preparation is warranted. Although these considerations will be discussed in Section 5, Experimental Elements, the use of freshly harvested versus frozen virus will be discussed here, given the underlying principals are physical characteristics of the virion proper. In the case of ZIKV, the effect of frozen virus in the preparation of infectious bloodmeals has been demonstrated in two separate analyses to reduce the infectivity of *Ae. aegypti* [54,66]. Specifically, long-term freezing (defined as viral stocks frozen for greater than one week) of ZIKV PRVABC59 (human isolate, Puerto Rico, 2015, hereafter referred to as ZIKV-PR) was found to infect *Ae. aegypti* (Poza Rico, Mexico, F11–13) less efficiently as assayed 7 and 14 days post-oral exposure compared to freshly harvested virus, or virus that was frozen for only 4 hours prior to use in bloodmeal preparation as determined by a cell culture-based infectious assay. The 7 days post-oral exposure time point also demonstrated that even a short-term freeze adversely affected the ability of ZIKV to disseminate compared to fresh virus, although this phenotype was not seen 14 days post-oral exposure [54]. This effect of freezing was independently verified in *Ae. aegypti* from La Plata, Argentina (F7–F8), in which a freshly cell culture-harvested ZIKV-PR at a titer of ≈9.1 log_10_ PFU/mL was found to infect (96% versus 16%), disseminate (88% versus 0%), and transmit (52% versus 0%) more efficiently than the same stock after freezing [66], as determined by real-time polymerase chain reaction (RT-PCR) mosquito assays. This phenomenon is not limited to ZIKV; previous work on a YFV isolate from a human patient in Peru (1899/81) was utilized to orally infect *Ae. aegypti* from Rexville, Puerto Rico. Virus was propagated in BHK-21 cells, clarified, and either frozen at −70 °C or held at 4 °C. Frozen stocks were thawed and diluted alongside fresh virus to encompass a range of titers (≈5, 6, and 7 log_10_ PFU/mL); then, they were mixed with washed human erythrocytes, and engorged mosquitoes were incubated for 12 days prior to immunofluorescence assays. At each tested titer, both infection (96% versus 63%; 44% versus 15%; 17% versus 0% at 7, 6, and 5 log_10_ PFU/mL, respectively) and dissemination (52% versus 20%; 28% versus 2%; and 8% versus 0% at 7, 6, and 5 log_10_ PFU/mL respectively) were significantly higher in the fresh virus condition, despite a relatively “short-term” freezing of viral stocks [67]. This general finding was corroborated dramatically in a separate analysis of both St. Louis encephalitis virus (SLEV) and DENV-2 in *Cx. quinquefasciatus* and *Ae. aegypti*, respectively. These studies revealed that frozen SLEV failed to infect or disseminate in over 100 mosquitoes, while the fresh equivalent infected 97% of mosquitoes and was disseminated in 78%; frozen DENV-2 only infected 1 out of 100 mosquitoes and failed to disseminate, while the fresh virus infected 56% of *Ae. aegypti* and was disseminated in 30% of infected individuals [68]. Finally, while the disparity between the infectivity of fresh versus frozen virus is most widely reported for flaviviruses, Rift Valley fever virus (RVFV) (Family *Phenuiviridae*, Genus: *Phlebovirus*) showed similar trends in both *Ae.* (*Ochlerotatus*) *taeniorhynchus* and *Cx. pipiens* mosquitoes, with both mosquito species becoming more efficiently infected when taking a bloodmeal from viremic hamsters, compared to pledgets soaked in the fresh or frozen blood from the same hamsters [69]. 

Despite the large body of evidence supporting the higher infectivity of freshly harvested virus for arthropod infection in multiple mosquito/virus combinations, this finding is not universal. In an analysis of ZIKV (strain TS17-2016), two populations of *Ae. aegypti* (Townsville and Innisfail, Queensland, F3–F5 and F2–F3, respectively) were exposed to both freshly (7.7 log_10_ 50% tissue culture infectious dose (TCID_50_)/mL) or frozen ZIKV (7.2 log_10_ TCID_50_/mL). Even considering the negligible difference in titer, neither mosquito population demonstrated significant increases in infectivity (Townsville fresh: 0% versus Townsville frozen: 10%, Innisfail fresh: 17% versus Innisfail frozen: 18%), dissemination (Townsville fresh: 0% versus Townsville frozen: 10%, Innisfail fresh: 17% versus Innisfail frozen: 12%), and transmission (Townsville fresh: 0% versus Townsville frozen: 10%, Innisfail fresh: 11% versus Innisfail frozen: 6%) [70]. It is worth noting that in this experimental paradigm, frozen virus and fresh virus were generated from disparate stocks, with the frozen preparation undergoing concentration prior to freezing, underscoring the profound effect exerted by disparities in methodologies. Given the wide range of methodologies utilized in laboratory vector competence analysis, it is important to consider the preparation of the viral stock utilized for experimental bloodmeals, especially in the context of standardization.

### 3.3. Use of cDNA Clone-Generated Arboviruses in Vector Competence Experiments. 

A common methodology of studying arboviruses is the use of reverse genetic systems. This technology allows for the cultivation of stock viruses based on defined consensus sequences, negating the necessity for subsequent cell culture passage (discussed below). Although several ZIKV full-length infectious clones (FLIC) exist, for the purposes of this discussion, three will be examined. The first of these was based on the Asian lineage ZIKV FSS13025 (human isolate, Cambodia, 2010), hereafter referred to as ZIKV-FLIC^FSS^. Clone-derived virus demonstrated some differentiation from the parental isolate, first and foremost being a homologous small plaque phenotype, compared to the heterologous plaque sizes seen in the parental strain, which were hypothesized to be due to the restriction of the clone to the homogenous population of virus born from the viral consensus, as opposed to the genetic heterogeneity present in the parental isolate [71]. Similar alterations were noted in mouse virulence (delayed disease course) and mosquito infectivity (increased infectivity and dissemination compared to parental virus), which were also posited to be due to the restriction of genetic diversity from the cloning strategy employed [71]. Likewise, the ZIKV infectious clone system based on the 2015 isolate from Paraiba Brazil (called ZIKV-ICD) demonstrated significant attenuation in multi-step replication kinetics in seven of nine tested cell lines (Vero, C6/36, human foreskin fibroblasts, SH-SY5Y, BeWo, HTR-8/SVneo human trophoblasts, and JAR human placenta cells), compared to the parental virus [72]. Deep sequencing analysis demonstrated a 2.3-fold increase in overall mutational frequency in WT ZIKV-Paraiba compared to ZIKV-ICD, with 61 nucleotides in the wild-type demonstrating polymorphisms at a frequency of over 1%, while ZIKV ICD demonstrated only a single nucleotide with a polymorphism (1.37%) [72]. Finally, the infectious clone system made for the American outbreak strain ZIKV PRVABC59 (human isolate, Puerto Rico, 2015), hereafter referred to as ZIKV FLIC^PR^, performed comparably to the parental isolate in various outputs, including growth kinetics (Huh7, JAR, Aag2, Vero, SH-SY5Y, NTERA2 cI.D1 C6/36, and BHK), mosquito infectivity, and animal pathogenesis. However, when examined for genetic diversity, ZIKV FLIC^PR^ proved less rich in single nucleotide polymorphism (SNP) diversity in several measures (distance, complexity, and nucleotide diversity) compared to the parental strain [73]. While undoubtedly an invaluable tool, it is therefore critical to consider the advantages and disadvantages of utilizing infectious clones as opposed to arbovirus isolates. However, even in experimental paradigms where infectious clones may not be entirely appropriate, they may be the only option available to investigators, as ready access to relevant isolates is not uniform. 

### 3.4. Passage History of Arboviruses Used for Vector Competence Assessment

Perhaps the most critical point to consider in the design of vector competence experiments from a virological perspective is the passage history of the arbovirus in question. It is well-documented that repeated cell culture or animal passage of a virus can dramatically alter its phenotype [74]. While this phenomenon has not been directly tested for ZIKV (i.e., identical strains with differing passages being subsequently analyzed for vector competence), the initial description of ZIKV from the late 1940s utilized multiple mouse brain passages, which adapted ZIKV from merely sickening the animals to producing neurologic symptoms and lethality following 17 mouse brain passages [1,2]. A more direct correlate of passage history with mosquito infectivity was demonstrated via the comparative analysis of four ZIKV strains (MR766, ZIKV-PR, ZIKV-FLR (Columbia, 2015), and ZIKV-PAN (Panama, 2015)) that had been amplified on either Vero or C6/36 cells. Utilizing multi-step replication kinetics, all four viruses reached peak titers more quickly when the virus used was derived from the same cells used for infection (mammalian versus C6/36 mosquito cells) [75], suggesting a potential trade-off in fitness for mammalian versus arthropod hosts. In another study, in-depth analysis of ZIKV-PR provided even more evidence supporting the major effect of passage history; two nonsynonymous mutations (E-V330L and NS1-W98G) were identified in Vero passage 3 viral stocks as well as ZIKV FLIC^PR^ that were not present in the original patient’s serum [76]. These mutations were associated with the attenuation of ZIKV infection of AG129 (mice lacking receptors for α/β/γ interferons), with the E-330L mutation alone significantly increasing median survival time and causing reduced weight loss compared to the re-engineered wild-type virus (E-330V/NS1-98W). Follow-up analysis via competition assay in Vero cells where wild type (E-330V/NS1-98W) was mixed in a 10:1 ratio with either the single (E-330L/NS1-98W) or double mutant (E-330L/NS1-98G) demonstrated that the single mutant and wild type persisted through three cell culture passages, while the double mutant began to outcompete the wild type by the third passage [76]. Examples from DENV, SLEV, WNV, and VEEV arboviruses also support a major role for passage history in the outcome of vector competence evaluations. Both a sylvatic (P8-1407) and endemic (IQT-1950) strain of DENV-2 that underwent 10 passages in a human liver cell line (Huh7), or in C6/36 mosquito cells, or that underwent alternating passages between these two cells showed adaption to the cell line used for passage with decreased fitness in the bypassed cell line, compared to the parental virus. In contrast, alternately passaged viruses demonstrated either fitness gains in both cell lines [77]. Work on SLEV and WNV utilized serial passages in C6/36 mosquito cells that bypassed an avian cell line (chicken embryonic fibroblasts; DF-1), found both viruses demonstrated enhanced replication in C6/36 cells, following serial passage in that cell line when compared to unpassaged virus, with no significant differences observed in kinetics on DF-1 cells for either virus [74]. The use of competition assay to determine relative fitness supported these results, as both viruses demonstrated increases in C6/36 replication following serial passage, although in this modality, C6/36-passaged viruses also demonstrated some decreases in fitness in the bypassed DF-1 cells [74]. Similar work conducted on VEEV (ID and IC serotypes) utilized an in vivo passaging strategy (mammalian: mouse or hamster, arthropod: *Ae. aegypti* mosquitoes) with fitness losses and gains determined via mosquito infectivity and mammalian viremia. Unsurprisingly, p10 viruses that passaged exclusively in mosquitoes demonstrated decreased viremias in their respective mammalian host when compared to the parental virus, while simultaneously increasing in mosquito infectivity. Reciprocally, p10 viruses passaged solely in mammals demonstrated increased peak viremias while decreasing in mosquito infectivity. Both ID and IC viruses, when passaged alternately between mammalian host and mosquito vector, exhibited no significant increases or decreases in fitness in either system when compared to parental viruses [78]. Therefore, it is quite clear that in an ideal setting, investigators should strive to utilize viral strains with minimal cell culture passages, or perhaps avoid those passaged multiple times on a single cell line; alternating vertebrate and insect cells may be a good compromise. In the case of vector competence work conducted in the context of ZIKV for example, the use of the MR766 prototype strain (which underwent over 140 suckling mouse brain passages [2,79]) may not be entirely appropriate to use to draw conclusions about more contemporary strains with a shorter passage history.

## 4. Mosquito/Entomological Determinants of Vector Competence

The role of vector mosquitoes in the design and implementation of vector competence experiments cannot be understated. Conversely, many of the parameters considered herein are more difficult and/or labor intensive to control, as investigators may be limited by mosquito populations available locally and in the laboratory. Herein, we consider the roles of mosquito species/genus/population, colonization, microbiome, and immune status, as well as insect-specific viruses on vector competence.

### 4.1. Mosquito Genus and Species. 

Many vector competence studies have focused on *Ae. aegypti aegypti* (the urbanized subspecies of *Ae. aegypti*, compared to *Ae. aegypti formosus*, the ancestral form restricted to sylvan biomes of West Africa) [80], which has been partially due to the ease of rearing these mosquitoes in laboratory settings [27], as well as the confirmed role of *Ae. aegypti aegypti* in the transmission of epidemic viruses DENV, YFV, CHIKV, and ZIKV. Unsurprisingly, the vast majority of ZIKV vector competence literature focuses on *Ae. aegypti* (reviewed [27,81,82,83]). The conclusion from all these analyses is that there is substantial variation in susceptibility to ZIKV across *Ae. aegypti* populations, with variations within populations observed across various viral strains [27]. However, given the explosive spread and breadth of the 2015–2016 ZIKV outbreaks, coupled with recent evidence of vector-adaptive mutations facilitating CHIKV adaption to a novel vector (CHIKV E1 A226V [84], followed by independent secondary E2 mutations such as L210Q or K252Q [85,86], favoring *Ae. albopictus* transmission), substantial effort has been expended on alternative vectors. ZIKV transmission competence has been demonstrated in *Ae. albopictus* [28,66,87,88,89], *Ae. vexans* [90], *Ae. notoscriptus* [91,92], *Ae. camptorhynchus* [91], *Ae. vitattus* [93], *Ae. luteocephalus* [93], and *Sabethes cyaneus* [94]. In addition to these species, the role of *Culex* spp. mosquitoes, in particular *Cx. quinquefasciatus*, which is often the most abundant tropical urban species, remains controversial. Field evidence suggesting the potential infection of this vector [95,96] initiated substantial efforts to examine *Cx. quinquefasciatus* by means of vector competence analysis. Despite this early evidence, the vast majority of the vector competence literature that has been published to date do not support the ability of this mosquito to become infected and transmit ZIKV [97,98,99,100,101], with no robust field evidence supporting its role during outbreaks [95,96,102]. However, it cannot be ruled out that only specific virus/mosquito population combinations allow for effective transmission. It is also worth noting that some of these analyses are not conducted with mosquito species in the range of ZIKV circulation, e.g., *Ae. notoscriptus* and *Ae. camptorhynchus*, which were used to determine if Australian mosquitoes have the potential to transmit ZIKV [91,92]. Likewise, *Sabethes cyaneus* has never been implicated as a vector of any arboviral pathogen [94], but served as a colonized proxy for other sylvatic *Sabethes spp.* mosquitoes to assess the potential for ZIKV to spill back into a sylvatic cycle in the Americas [7,9,94]. 

Experiments investigating the competence of mosquito genera and species not commonly associated with the transmission of a given arbovirus must likewise be grounded in ecologic relevance. For example, with respect to vectorial capacity, a mosquito species that is transmission-competent for a given agent but does not exhibit a tendency toward biting humans (whether because it does not live in areas populated by humans, or as a matter of host preference) is unlikely to play a role in urban transmission cycles of human-amplified viruses. Therefore, in such a situation, experiments conducted on this hypothetical vector would require clear communication of the rationale and hypotheses.

### 4.2. Effect of Mosquito Colonization on Vector Competence. 

As previously indicated, the abundance of vector competence experiments using *Ae. aegypti* reflect its ease of rearing and colonization in laboratory settings [27]. Indeed, commonly used laboratory “reference” strains exist, such as the Rockefeller (ROCK) colony, Higgs White Eye (HWE), and Rexville (RED), with the former established in Cuba in 1930 [103,104]. The widespread use of these lines underscores the concern that long-term colonization may impart phenotypic and genotypic changes, some of which can modulate competence. This has been empirically demonstrated in a comparative analysis of field-derived populations of *Ae. aegypti* from Iquitos (Peru, F2) and McAllen (Texas, US, F3), which were compared to ROCK *Ae. aegypti* for their competence for DENV-2 from either Southeast Asia or the Americas. Both field populations demonstrated increased susceptibility to disseminated infection by the Southeast Asian DENV-2 strains, compared to American DENV-2 (McAllen: 81% versus 67%, Iquitos: 69% versus 41%), while the ROCK *Ae. aegypti* exhibited no significant difference in susceptibility between virus strains (SE Asia: 60%, American: 67%) [105]. Although the mechanistic underpinnings of how colonization affects a given mosquito population’s susceptibility to infection are not fully understood (although growing evidence indicates a role for the microbiome, as reviewed below), colonization has been reported in the literature for decades to affect vector competence. In an analysis of Egyptian *Cx. pipiens*, the susceptibility to infection by, and transmission of RVFV was compared across 16 mosquito generations in laboratory settings. While susceptibility to infection increased with colonization (F1 infection rate = 67%, F16 infection rate = 84%, *y* = 58.1 + 0.94x, *r* = 0.67, *p* < 0.001), transmission competence decreased (F1 transmission rate = 48%, F16 transmission rate = 21%, *y* = 41.3 − 1.0x, *r* = −0.76, *p* < 0.001) [106]. Similar work conducted on an *Ae. aegypti* colony from Vero Beach, Florida, which also demonstrated an overall decrease in disseminated YFV infections with colonization (F1 = 54.5%, F10 = 12%) [107]. While there is undoubtedly value to be derived from utilizing widely available reference strains (e.g., ROCK, HWE, RED, etc.), these studies highlight that laboratory-colonized mosquitoes do not necessarily reflect their feral counterparts, underscoring the value of vector competence studies with relevant field populations of vector mosquitoes as well. 

### 4.3. The Mosquito Microbiome 

In recent years, the role of the mosquito microbiome in vector competence has gained increasing attention. Mosquitoes are colonized with a multitude of microbial gut flora such as bacteria and fungi early in development [108]. The mosquito microbiome can be influenced by that of its parents (vertical transmission), sex, larval environment, life stage, geographic origin, etc. It is increasingly understood that an individual mosquito can be considered a single holobiont unit, where the mosquito, its microbiome, and a given arbovirus maintain a reciprocal tripartite interaction [109]. The relationship between the mosquito microbiome and arboviruses is largely mediated via the mosquito’s innate immune system, which is hypothesized to be stimulated to a basal level of activation by the presence of the microbiome [109]. Here, we review the midgut microbiome and its role in arbovirus vector competence and mosquito immunity, although this is by no means an exhaustive treatment, as mosquitoes contain microbiota associated with other discrete organs/tissues (e.g., salivary glands, hemocytes, reproductive tissues) [108,110]. The interactions between the mosquito, microbiome, and arbovirus vector competence are especially relevant in the context of the above discussion of laboratory colonization. A recent study utilized 16S sequencing to determine the diversity, abundance, and community structure of the midgut microbiome of six geographically distinct *Ae. aegypti* colonies (Australia, Cambodia, French Guiana, Gabon, Guadeloupe, and Uganda) of relatively low generations (≤F10), and demonstrated striking similarities among all [111]. Along with other analyses demonstrating that field caught (F0) mosquitoes (*Ae. aegypti*, *Ae. albopictus*, and *Cx. quinquefasciatus*) have disparate microbiome constituents compared to their colonized counterparts [112], these findings suggest that the laboratory colonization of mosquitoes can inadvertently alter their microbiomes. With respect to the effects of the microbiome on vector competence, substantial work has been conducted utilizing *Ae. aegypti* and DENV-2. For example, in an elegant study conducted on ROCK *Ae. aegypti*, the role of microbiome in vector competence was demonstrated via the comparison of midgut titers of conventionally raised mosquitoes and aseptic mosquitoes in which the microbiome was depleted via a sterile housing and antibiotic cocktail treatment post-eclosion. This analysis demonstrated that, compared to their aseptically raised counterparts, conventionally reared mosquitoes demonstrated elevated levels of several Toll pathway-regulated antimicrobial peptides (defensin, cecropin, attacin, and gambacin) and had two-fold lower DENV-2 titers in the midgut [113]. Additional analyses demonstrated that silencing Cactus via siRNA (thereby transiently activating Rel 1, a transcription factor regulating transcription of Toll effectors) was associated with a four-fold reduction in midgut titers compared to control (GFP-dsRNA treated) mosquitoes. The contribution of the Toll pathway was further demonstrated by siRNA silencing of the upstream adaptor MyD88, inhibiting the activation of downstream Toll pathway elements and reducing by >2.5-fold DENV-2 midgut titers, while silencing the adaptor Caspar of the IMD pathway failed to significantly alter midgut titers. These results highlight the functional importance of the Toll pathway in attenuating DENV infection [113]. As an emerging field within the vector competence literature, the role of the microbiome on the mosquito innate immune pathways and the consequences for vector competence are wide and varied. Individual bacterial species (*Chromobacterium Csp_P*, *Proteus*, *Paenibacillus,* and *Wolbachia*) have been associated with decreasing the transmission competence of *Ae. aegypti* for DENV-2 [109,110,114], while in other analyses, *Wolbachia spp*. and *Serratia spp*. have been demonstrated to increase the transmission of WNV (by *Cx. tarsalis*) and DENV-2 (*Ae. aegypti*) [115,116]. Even more critically, given the role of the microbial flora in the basic physiology of individual mosquitoes (e.g., nutrient exchange, immunity, longevity, and reproduction) [109,117], there could be major effects on vectorial capacity. As such, the role of the microbiome should receive increased attention in analyses of vector competence, and manipulation of the microbiome to alter competence could be a promising strategy for the control of arboviral diseases. Although standardization of the microbiome would be ideal, but is probably not feasible in most laboratories, samples should at least be collected from mosquito populations for which vector competence is reported in the literature.

### 4.4. The Mosquito Virome (Insect-Specific Viruses)

Similar in principal to the role of the microbiome in vector competence, the role of the vector virome also deserves special consideration. The role of insect-specific viruses (ISVs) in affecting vector competence should be considered, as well as their potential use in reducing transmission. Most ISVs have been detected in mosquito samples, which is due in part to sampling bias in arbovirus surveillance, as well as the existence of a susceptible mosquito cell line (C6/36, *Ae. albopictus*) carrying a defective RNAi response [118,119]. To date, insect-specific viruses have been discovered as members of the order Bunyavirales, and the families *Flaviviridae*, *Togaviridae*, *Reoviridae*, *Rhabdoviridae*, and *Mesoniviridae*, and the proposed taxon *Negevirus* [118,119,120,121], of which the latter two are comprised solely of ISVs. These viruses are often detected during arbovirus surveillance studies upon the screening of mosquito samples in insect cell cultures [122]. Despite their intrinsic host restriction, often limiting their ability to infect and/or replicate in vertebrate cells, these agents are often closely related to pathogens of clinical and veterinary importance [118,119,120]. For example, Eilat virus (EILV), an alphavirus within the *Togaviridae* family that is a sister clade to the western equine encephalitis virus (WEEV) serocomplex, was originally isolated from a pool of *Anopheles coustani* mosquitoes from the Negev desert near the city of Eilat region in Israel [122,123]. EILV is restricted to replication in arthropods/mosquitoes at both the viral attachment/entry as well as the genomic replication levels, and exhibits limited ability to orally infect mosquitoes. It cannot infect *Ae. albopictus* at a bloodmeal titer of 9.0 log_10_ PFU/mL; it only infects *Cx. quinquefasciatus* and *An. gambiae* with this same high titer, but can infect *Ae. aegypti* at oral doses of 7.0 log_10_ PFU/mL [124]. EILV induces the homologous and heterologous interference of superinfecting alphaviruses in both insect cell culture as well as during in vivo infections of *Ae. aegypti* with CHIKV. Specifically, the pre-infection of C7/10 *Ae. albopictus* cells with EILV 24 hours prior to superinfection with either VEEV (TC-83) or Sindbis virus (SINV) significantly decreases titers of the superinfecting virus and delays peak replication [123]. Similarly, intrathoracic (IT) infection of *Ae. aegypti* with EILV 7 days prior to oral exposure to CHIKV decreases the infection rate observed at 3 and 5 days post-infection (DPI) compared to PBS-inoculated controls, although this decrease disappears by 7 DPI [123]. In the case of insect-specific flaviviruses (ISFs), there exist two distinct clades, one which lies well outside the clade of arboviruses. This group includes viruses such as cell fusing agent virus (CFAV), Culex flavivirus (CxFV), and Kamiti River virus (KRV), while the other clades lie within the mosquito-borne arboviruses as close relatives of Japanese encephalitis virus (JEV), DENV, ZIKV, and WNV, among many others [119,125]. One in the latter group, called Nhumirim virus (NHUV), has also demonstrated an ability to interfere with infection by heterologous flaviviruses, reducing replication kinetics in C6/36 cells of SLEV, JEV, and WNV whether the second virus was inoculated simultaneously (co-infection), or superinfected 1, 3, or 5 DPI [126]. This effect was reproduced with ZIKV and DENV-2, and IT inoculation of NHUV into *Ae. aegypti* 6 days prior to a ZIKV-infectious bloodmeal caused a significant decrease in ZIKV infection and dissemination. The co-inoculation of both ZIKV and NHUV to *Ae. aegypti* significantly decreased ZIKV transmission in saliva compared to mosquitoes solely inoculated with ZIKV [127]. Similar observations have been made in *Ae. aegypti* infected IT with CFAV. Specifically, *Ae. aegypti* were IT inoculated with CFAV two days prior to oral exposure to either ZIKV (PF2013, French Polynesia, 2013) or DENV-1 (KDH0026A, Thailand, 2010), and heads were removed and tested independently for both the presence of virus and titer 2, 5, 7, and 13 DPI [128]. Although there were no differences in the rate of DENV infection between CFAV-infected and mock-infected mosquitoes, both the rate of dissemination and the titer of DENV present in the head were significantly lower in CFAV-infected mosquitoes when infectious output assays were conducted in C6/36 mosquito cells. Similar results for DENV-1 were observed in an identical experimental paradigm utilizing infectious assays conducted on Vero cells (to preclude CFAV-induced cytopathic effects in C6/36 cells as a confounding element). Strikingly, ZIKV infection and dissemination rates were unaffected by prior CFAV infection (only assayed in Vero cells), although head titers 13 DPI were significantly lower in CFAV-infected *Ae. aegyti* [128]. Given that infection with ISVs has been demonstrated to alter kinetics of arboviral infection in vitro and in vivo, their presence in established mosquito colonies likely acts as a confounding variable in competence analyses. Indeed, in an analysis conducted on 16 established laboratory colonies of mosquitoes, three were found to be persistently infected with ISVs [127], underscoring the necessity of the routine screening of laboratory populations for ISVs.

## 5. Experimental Variables in Vector Competence Assessment

In addition to parameters that are intrinsically vector-driven or virus-driven, vector competence experiments include a multitude of variables in experimental design. A great many of these arise from the inherent conflict that arises from attempting to capture the entirety of complex biological systems with experimental rigor and control, resulting in compromises between the standardization of methods and ecological relevance, and sometimes the requirements for arthropod containment. Herein, we consider the roles of bloodmeal dosage, bloodmeal type (artificial versus live, compositions of artificial bloodmeals, effects of anticoagulants, etc.), sequential blood feeding, and ambient temperature during extrinsic incubation.

### 5.1. Bloodmeal Titer and Viremia. 

One of the most important determinants of mosquito infectivity, and subsequently transmission, is the orally available dose (i.e., the bloodmeal titer), or as described by Kuno and Chang, “Theoretically, the higher the concentration of virus in the blood and the longer the duration of viremia, the greater the probability of a vector acquiring a virus from infected vertebrates” [129]. Unsurprisingly, the vast majority of laboratory vector competence data support this finding in multiple combinations of virus species and mosquito species. With respect to ZIKV, multiple populations of *Ae. aegypti* and *Ae. albopictus* have demonstrated increasing levels of infection, dissemination, and transmission when exposed to increasing oral doses [28,29,130], as has *Ae. notoscriptus* for infection (however, not transmission) with ZIKV strain MR766 [92]. Likewise, the vector competence of various *Culex spp.* mosquitoes for WNV is highly dependent on oral doses [131,132]. Despite these repeated findings, the importance of viremia magnitude should be interpreted with caution, as the data from many of these ZIKV analyses suggest that this virus requires oral doses that are higher than those indicated by human viremia estimates [133,134]. This is well illustrated in the recent work conducted in Iquitos, Peru, in which naturally DENV-infected volunteers allowed naïve *Ae. aegypti* to feed directly, or exposed mosquitoes to feed indirectly, on these doses via venipuncture, and the use of anticoagulated blood or unmanipulated blood for artificial bloodmeals. A subset of the volunteer population demonstrated no detectable viremia, yet were still capable of infecting *Ae. aegypti* (direct bloodmeals: 8/18, 44%, indirect anticoagulated bloodmeals: 7/14, 50%, and indirect bloodmeal with no additives: 6/20, 30%) [135]. Similar work in nonhuman primate models of both DENV and ZIKV infection have shown direct oral infection of *Ae. aegypti* at low to undetectable titers (DENV-2: undetectable to 3.3 log_10_ PFU/mL; ZIKV: 4.4 log_10_ genome copies/mL) [30,136]. Given that fully engorged mosquitoes generally imbibe 5–10 μL of blood [137], a bloodmeal containing 3.0 log_10_ infectious units per milliliter would contain few if any infectious virions, assuming a random distribution of virus throughout the circulation [138]. Considered in the context of previous work in the VEEV/*Ae. taeniorhynchus* system, which illustrated that only ca. 100 midgut epithelial cells are susceptible to infection, and often infection is initiated in 1–5 cells [139], these results suggest that a large proportion of wild mosquitoes feeding on viremic hosts fail to become infected and perpetuate transmission cycles. The roles of only infrequently transmission-competent infections—offset by huge numbers of transmission chains, persistent vertebrate infections, or vertical transmission in vectors—in arbovirus maintenance deserve more investigation.

### 5.2. Elements of Artificial Bloodmeals.

As reviewed above, laboratory vector competence experiments using artificial bloodmeals often lead to the conclusion that very high titers are needed to infect mosquitoes [140]. However, a substantial body of evidence across multiple arboviral families/orders (*Flaviviridae, Togaviridae*, and Bunyavirales) indicates that artificial bloodmeals are less infectious compared to bloodmeals taken from a viremic animal at equivalent titers [29,69,138,140,141,142,143,144]. Artificial bloodmeal methods vary widely among laboratories, but the vast majority are conducted utilizing either hanging blood drops, pledgets (cotton soaked in blood/virus mixtures), or membrane feeding systems [144], although the former two have seen less use in recent years. Membrane systems consist of heated (generally 37 °C) reservoirs filled with a blood and virus mixture [145]. Additional sources of variation include the membranes overlaying the reservoirs, which include animal skin, sausage casing, or laboratory films based on collagen or that are completely artificial [145,146], as well as the kind of blood utilized (animal versus human, whole blood versus washed erythrocytes, defibrinated or anticoagulated) [146,147,148]. The effect on vector competence of the type of membrane overlaying blood reservoirs does not appear to be an active area of research, other than the effect on feeding efficiency [146]. Surprisingly, there has also been scant attention to the effects of anticoagulants on vector competence results, and the few studies that have been conducted used different virus/mosquito combinations. Knox et al. noted that DENV-4 lost significant titers over the course of an hour in an artificial membrane feeder when using lithium heparin-treated rabbit blood, necessitating the use of sodium citrate [149]. Surprisingly, such a finding was not corroborated within the same study in the context of DENV-2, illustrating that anticoagulant effects may be highly species-specific and possibly even virus strain-specific. Mahmood et al. found no significant differences in the feeding efficiency or infection rates of *Cx. tarsalis* with WEEV in either heparinized or defibrinated rabbit or chicken blood [146]. These limited findings indicate the potential benefit to the vector competence field of further anticoagulant studies to determine the effects on feeding efficiency and arbovirus infectivity. Another source of variance in vector competence assessments is the source of blood used. Among the more widely used blood sources are sheep, human, bovine, chicken, and rabbit [150,151]. Although humans, chickens, and sheep are ecologically relevant with respect to some mosquito-borne arboviruses (e.g., DENV/ZIKV/CHIV, WNV/WEEV, RVFV), many studies utilize blood derived from vertebrates that are irrelevant to the mosquito species or arbovirus in question. As with many variables discussed here, the exact role played by blood source on the outcome experimental infections is largely unknown. When examined in the context of the presence/absence of anticoagulants, and the various bloodmeal compositions (e.g., washed erythrocytes versus whole blood), the potential variation from lab to lab becomes potentially enormous, illustrating the need for further research on an improved standardization of methods for vector competence research. 

### 5.3. Sequential Bloodmeals. 

Given the role of *Ae. aegypti* in the transmission of many of the most important epidemic arboviruses such as ZIKV, DENV, YFV, and CHIKV [9,60,152], it is not surprising that substantial efforts have been expended to examine multiple populations of this mosquito. Unfortunately, its widespread use in laboratory vector competence experiments and the need for tightly controlled protocols often ignores critical elements of *Ae. aegypti’s* natural behavior, some of which are paramount to its role as an efficient vector. Of these, the tendency of this mosquito to take multiple bloodmeals in a single gonadotrophic cycle [153,154,155,156] is perhaps the most important for enhancing vectorial capacity through multiple host contacts to enhance infection, and to increase transmission to multiple susceptible individuals [156,157]. Despite this well-known trait of *Ae. aegypti’s* biology, laboratory vector competence experiments typically involve mosquitoes that fully engorge on infectious blood, and then are maintained on sucrose over the course of the EIP. Armstrong et al. recently demonstrated the role of multiple bloodmeals on the vector competence of *Ae. aegypti* for ZIKV, DENV-2, and CHIKV. By exposing mosquitoes to a second, noninfectious bloodmeal 3–4 days following feeding on infectious blood [158], they showed that dissemination was significantly increased for all three viruses compared to mosquitoes that were not given the second noninfectious bloodmeal. The second bloodmeal did not affect initial infection, and its effect disappeared by 10 days of ZIKV extrinsic incubation. Nevertheless, the observed decrease in the EIP could have a major impact on vectorial capacity, since the EIP is treated exponentially by the equation discussed above. This finding highlights how laboratory competence assessments should strive to capture as many elements as possible of field conditions. 

### 5.4. Extrinsic Incubation Temperature

In addition to the effects of bloodmeal conditions and mosquito behavior on vector competence assessments, environmental factors also have major impacts. The role of ambient temperature on both vector competence and vectorial capacity (e.g., survival, and adult density via the effect on larval development [27]), is well documented. For example, an analysis of field-derived *Ae. aegypti* exposed to ZIKV and reared at 16 °C, 20 °C, 24 °C, 28 °C, 32 °C, 34 °C, 36 °C, or 38 °C ± 0.5 °C showed differences in infection, dissemination, and transmission rates [130]. Infection was least affected by temperature, with at least minimal rates observed at 16 °C (6%) and 38 °C (7%), with the highest efficiency in the 24–34 °C range (75–89%). Dissemination had a more limited optimal range with minimal efficiencies observed at 16–20 °C (3–4%) and 38 °C (5%), while maximum efficiency was observed between 28–34 °C (65–77%). Transmission capability demonstrated the narrowest range of efficiency with minimal rates observed between 16–24 °C (0–4%) and 36–38 °C (5–0.4%) and maximum efficiency within the 28–34 °C range (19–23%) [130]. Compared to ZIKV, CHIKV demonstrated an increased infectivity of *Ae. aegypti* Liverpool when mosquitoes were maintained at 18 °C compared to 28 °C, while YFV demonstrated increased infectivity in field-derived *Ae. albopictus* when the mosquitoes were maintained at 18 °C compared to 28 °C [159]. Conversely, DENV-2 is more infectious for two populations of *Ae. aegypti* (Nairobi and Kilifi) when the mosquitoes are held at a higher temperature range (29–31 °C compared to 25–28 °C), although the effect is more pronounced in the Nairobi population [160], indicating the possibility that the effect exerted by temperature may interact with the mosquito population or possibly the viral strain as well [161]. These varied results underscore the difficulty in predicting precisely the effects of climate change on the vector competence of natural mosquito populations.

## 6. Conclusions and Recommendations

While the ZIKV epidemic led to substantial strides in understanding vector competence, research has in some ways been hampered by a lack of concordance and standardization of methods. Despite many publications reporting the vector competence of various populations of potential vector mosquitoes using several strains of ZIKV, this lack of standardization precludes a large-scale meta-analysis on the subject. Due to continued increases in global trade/travel, the increasing urbanization of previously rural and sylvatic biomes, coupled with climate change, arbovirus emergence, and reemergence will undoubtedly continue to be a major public health challenge [61,162,163]. Given the major investments in ZIKV vector competence assessments, it would behoove arbovirologists to capitalize on ZIKV findings to begin discussions on how best to approach future research.

First and foremost, complete standardization is likely impossible, because it would place an undue burden on laboratories in more resource-poor settings. One avenue that has been proposed to improve comparisons among vector competence studies is the standardization of reporting methodologies [147] to increase both transparency and reproducibility. While establishing guidelines to encourage better reporting will undoubtedly benefit the field, some experimental standards would also be beneficial. For example, as described above, there is a substantial effect of oral dose (viral titer of the infected host) on the infection of a mosquito, especially in the context of artificial bloodmeals [129,138,140]. Ideally, vector competence assessments would therefore use viral titers that are consistent with viremia titers in humans or other amplification hosts. However, human arboviral pathogens may peak prior to the appearance of symptoms [164,165,166], which is complicated by the increasing use of PCR-based rather than infectious assays. Although the direct or indirect feeding of mosquitoes on the blood of infected persons with early diagnosis can mitigate this challenge [135], few research groups have access to this kind of study, which requires substantial community outreach and includes challenges to ethical approval [167]. For viruses with nonhuman amplification hosts, viremia estimates generally come from experimental infections, which are logistically challenging. In the absence of accurate human or other amplifying host viremia data, or human volunteer studies as described above, there is value to be gained from the development and utilization of animal models. In this, ZIKV provides an excellent example of the difficulties associated with models for several human arboviruses that do not replicate efficiently in immunocompetent mice [168,169,170]. Therefore, many of the developed models utilize mice that have had elements of their innate immune response altered (e.g., interferon receptor knockouts, human STAT2 knock-in, etc.) [168,171,172], rendering them even less relevant to natural transmission cycles. Therefore, the use of natural reservoirs as animal models would be ideal. However, in the case of several important arboviruses (YFV, DENV, CHIKV, ZIKV), the natural hosts are nonhuman primates [9,173], which require specialized facilities and expertise, and lack the same breadth of reagents compared to mice [168,174], not to mention the high costs when compared to murine models.

Despite some challenges with the standardization of experimental vector competence evaluations (e.g., lack of information on critical parameters, expense, access to resources), some elements that can more feasibly be standardized should be targeted. For example, given that ISVs are known to impact vector competence [123,127], it would not be unreasonable to expect investigators to routinely screen long-standing mosquito colonies for their presence via cell culture-based or PCR-based methodologies. Given that many vector competence analyses are performed in the context of local mosquito populations [29,88,91], and commonly used reference mosquito strains have existed for decades [103,104], often as disparate populations in various insectaries, the potential for variation in competence as a function of such ISVs should be ignored. The use of arbovirus strains that are unavailable to most investigators through repositories such as BEI, ATCC, or the World Reference Center for Emerging Viruses and Arboviruses (WRCEVA), should be carefully considered. Additionally, some degree of terminology standardization should be considered to avoid confusion in the literature [27]. For example, transmission competence should be used only to indicate the presence of virus in the saliva [31,34,35]. Virus detected in head squashes, or viral RNA detected in salivary glands [27], while suggestive, do not necessarily indicate transmission capability.

In summary, vector competence analyses, which are critical to arbovirology, involve complex and ever-evolving methods. Given the intersection of so many elements (entomology, virology, logistics/containment, etc.), it is not surprising that there is a substantial degree of variability in how these studies are conducted across labs. The ZIKV epidemic in the Americas illustrates how, despite the large amounts of data that have been generated, meta-analyses are currently not possible due to experimental variances. While outright standardization is currently impossible, the increasing calls for standardization in data reporting represent a springboard that the arbovirology community can utilize in some feasible approaches to increase both transparency and reproducibility in vector competence assessments.
